# Intravenous immunoglobulin fails to improve ARDS in patients undergoing ECMO therapy

**DOI:** 10.1186/s40560-018-0278-8

**Published:** 2018-02-26

**Authors:** Stefanie Prohaska, Andrea Schirner, Albina Bashota, Andreas Körner, Gunnar Blumenstock, Helene A. Haeberle

**Affiliations:** 1 Department of Anesthesiology and Intensive Care Medicine, Medical Faculty, University Hospital Tübingen, Eberhard-Karls University, Hoppe-Seyler-Strasse 3, 72076 Tübingen, Germany; 2 Institute for Clinical Epidemiology and Applied Biometry, Medical Faculty, Eberhard-Karls University, Silcherstraße 5, 72076 Tübingen, Germany

**Keywords:** Intravenous immunoglobulin, ARDS, Inflammation, ECMO

## Abstract

**Background:**

Acute respiratory distress syndrome (ARDS) is associated with high mortality rates. ARDS patients suffer from severe hypoxemia, and extracorporeal membrane oxygenation (ECMO) therapy may be necessary to ensure oxygenation. ARDS has various etiologies, including trauma, ischemia-reperfusion injury or infections of various origins, and the associated immunological responses may vary. To support the immunological response in this patient collective, we used intravenous IgM immunoglobulin therapy to enhance the likelihood of pulmonary recovery.

**Methods:**

ARDS patients admitted to the intensive care unit (ICU) who were placed on ECMO and treated with (IVIG group; *n* = 29) or without (control group; *n* = 28) intravenous IgM-enriched immunoglobulins for 3 days in the initial stages of ARDS were analyzed retrospectively.

**Results:**

The baseline characteristics did not differ between the groups, although the IVIG group showed a significantly reduced oxygenation index compared to the control group. We found no differences in the length of ICU stay or ventilation parameters. We did not find a significant difference between the groups for the extent of inflammation or for overall survival.

**Conclusion:**

We conclude that administration of IgM-enriched immunoglobulins as an additional therapy did not have a beneficial effect in patients with severe ARDS requiring ECMO support.

**Trial registration:**

Clinical Trials: NCT02961166; retrospectively registered.

## Background

Acute respiratory distress syndrome (ARDS) is characterized by pulmonary inflammation that can be caused by pulmonary and extrapulmonary origins. Sepsis, bacterial pneumonia, polytrauma, and aspiration pneumonia are the most common causes of ARDS [1]. Predictors of survival include age, the type of underlying medical condition, the severity of pulmonary injury, the presence of extrapulmonary organ dysfunction, and ongoing sepsis [2]. Currently, clinical attempts to rescue ARDS patients include individualized ventilation and fluid management, adequate infection control, including early application of broad-spectrum anti-infectives, neuromuscular blockade using cisatracurium, sedation strategies, prone positioning, and finally extracorporeal membrane oxygenation (ECMO) to ensure oxygenation [3–5]. Although the incidence of ARDS is relatively high, with five to eight cases per 100,000 European inhabitants and even more in the USA, the various pathomechanisms are only partially understood, resulting in different experimental approaches to understand immune responses during early ARDS [6].

One well-described issue is decreased immunoglobulin levels in patients with severe infection [6] as an element of the immunological response in the initial phase of inflammation in response to sepsis [7]. Therefore, one approach to support critically ill patients is intravenous administration of IgM-enriched immunoglobulins since this could potentially decrease the severity of inflammation. Although this treatment was omitted in recent sepsis guidelines due to a lack of supporting evidence in high-quality trials [8], several studies, including one meta-analysis, describe beneficial effects of immunoglobulins in acute pneumonia induced by drug-resistant bacterial infections [9–11]. Furthermore, several case reports describe beneficial effects of antiviral therapy in combination with intravenous immunoglobulin therapy in immune-compromised patients [12–14]. Based on these data, we treated patients with ARDS requiring ECMO therapy with IgM-enriched immunoglobulins immediately after intensive care unit (ICU) admission. The objective of this study was to investigate whether intravenous immunoglobulin administration could improve the clinical course of ARDS in patients treated with ECMO. Therefore, mortality, the duration of ECMO therapy, the incidence of renal replacement therapy, the duration of vasopressor and anti-infective therapy, length of stay in the ICU, and length of stay in the hospital were analyzed retrospectively in 57 ARDS patients requiring ECMO therapy.

## Methods

The study was approved by the local research ethics committee of the University Hospital and the Eberhard-Karls University Tübingen, Germany.

### Study patients

Patients with severe ARDS treated with ECMO therapy between January 2012 and January 2016 at our institution were analyzed retrospectively. In all patients, ECMO therapy was required due to hypoxia and/or increased pulmonary resistance precluding protective lung ventilation.

### Therapy

ECMO therapy was performed according to the guidelines of the Extracorporeal Life Support Organization (ELSO) using ILA-active Systems (Novalung, Stolberg, Germany [15]. Fifty-seven patients were analyzed. Twenty-eight patients were treated with IgM-enriched immunoglobulins (Pentaglobin®; Biotest; Dreieich, Germany) (IVIC-group). Pentaglobin is an IgM-enriched polyvalent immunoglobulin preparation derived from a plasma pool. It contains 6 mg of IgM, 6 mg of IgA, and 38 mg of IgG (63% IgG1, 26% IgG2, 4% IgG3, and 7% IgG4) per millilitre.

The indication for IgM-enriched immunoglobulin (IVIG) treatment was determined at the discretion of the treating intensivist. IVIG was applied if a viral infection or an infection due to multi-resistant gram-negative bacteria (MRGN) was suspected. IVIG dosing was performed according to the instruction of the manufacturer: 0.4 ml/kg/h (up to 100 ml) as the initial dose, followed by 0.2 ml/kg/h for 72 h until a total dose of 15 mg/kg was achieved. Twenty-nine patients did not receive immunoglobulins (control group).

Vasopressors (Norepinephrine; Arterenol® Sanofi-Aventis; Germany) were used after volume resuscitation according to the sepsis guideline (13). Renal replacement therapy was performed with citrate anticoagulation (Multifiltrate, Fresenius Medical Care, Bad Homburg v.d.H. Germany) in patients with acute renal failure.

Data were retrospectively extracted from an ARDS-specific database at our institution. Since the electronic patient management system was changed during this time frame, not all required data were available for all ARDS patients.

MRSA and VRE screening was performed routinely in all patients admitted to the ICU.

To identify causal infections, bronchoalveolar lavage (BAL) samples, blood cultures, urinary samples, and perioperative samples were analyzed. In patients at risk, PCR for atypical pathogens was also performed in BAL samples. Infection was defined as > 10^6^ colony-forming units (CFU) in BAL and/or urinary cultures. For blood cultures, any replicate of bacterial growth was defined as infection. During flu season, influenza infection was detected by PCR. BAL samples were also analyzed for other viral pathogens, such as cytomegalovirus (CMV) and herpes simplex virus (HSV). Positive findings for these viruses were confirmed by cell cultures. Patients with unknown pathogens and immune suppression were additionally screened for other pathogens.

Anti-infective therapy was applied according to the local guideline considering local resistance patterns.

### Statistical analysis

Categorical data are reported as numbers and percentages, and continuous data are summarized with the median and range (i.e., the minimum and maximum values) unless otherwise indicated. To compare categorical variables or outcomes, such as the occurrence of infection or in-hospital death, between the IVIG group (*n* = 28 patients) and the control group, which included 29 patients who did not receive IVIG therapy, the chi-squared test was used. For inter-group comparisons of continuous data, the two-tailed two-sample *t* test was generally performed. If the original data exhibited a log-normal distribution, e.g., ICU length of stay (LOS), then raw data were log-transformed prior to analysis with the *t* test. A *p* value < 0.05 was assumed to indicate a statistically significant difference between the groups. The data analysis was performed using JMP® 11.0 statistical software (SAS Institute Inc., Cary, NC, USA).

## Results

### Patients

A total of 57 patients with ARDS requiring ECMO therapy between 2013 and 2016 were analyzed retrospectively. Twenty-eight patients were treated with IgM-enriched immunoglobulins (IVIG) for 3 days according to the manufacturer’s instruction (IVIG group), and 29 patients did not receive IVIG therapy (control group). No adverse events were reported after IVIG application. The median age of both groups was 52 years, ranging from 27 to 76 years in the control group and from 17 to 78 years in the IVIG group. The median Simplified Acute Physiology Score (SAPS) and Acute Physiology And Chronic Health Evaluation (APACHE) score were comparable between both groups (Table 1). The median APACHE score in the IVIG group was 24 vs. 23 in the control group. In 30% (8/27) of the IVIG-treated patients and 31% (9/29) of the control patients, the APACHE score was equal to or greater than 28. Regarding preexisting disease, diabetes mellitus, cardiac disease, immune suppression, and malignancy were more common in the IVIG group (Table 1).

**Table 1:** Characteristics of ARDS patients included into the study

	ARDS patients without IgM-enriched IVIG treatment	ARDS patients with IgM-enriched IVIG treatment	P Value
Baseline Characteristics			
Number of patients	29	28	
Age [years: median/ min/ max]	52/ 27/ 76	51.5/ 17/ 78	
Female/male [number]	8/21	14/14	
Duration of IVIG therapy	0	3	
APACHE Score [median, min, max]	24/ 12/ 36	23/ 8.5/ 38	p=0.99
SAPS Score [median, min, max]	40/ 18/ 70	45/ 14/ 77	p=0.99
Preexisting diseases			
Heart disease [number;%]	4; 14%	7; 25%	
Diabetes mellitus [number;%]	3; 10%	6; 21%	
Hypertension [number;%]	10; 35%	10; 36%	
Immune suppression [number;%]	3; 10%	6; 21%	
Malignancy [number;%]	3; 10%	6; 21%	
Smoking [number;%]	10; 35%	5; 18%	
Obesity [number;%]	9; 31%	8; 29%	
ARDS			
PaO_2_/FiO_2_ [median/ min/ max]	93/ 41/ 253	67/ 43/ 162	p=0.009
PaO_2_/FiO_2_ ≤ 100 [number;%]	16; 55%	21; 75%	
PaO_2_/FiO_2_ >100≤ 200 [number;%]	10; 35%	7; 25%	
PaO_2_/FiO_2_ >200≤ 300 [number;%]	3; 10%	0	
Duration of Ventilation [Hours; median, min, max]	399/ 20/ 1323	538/ 91/ 2472	
Infection			
Multiresistant pathogen [number; %]	0	2; 7%	
Bacterial infection [number;%]	11; 38%	11; 39%	
Legionella Pneumoniae [number;%]	1; 3%	3; 11%	
Streptococcus pneumonia [number;%]	1; 3%	3; 11%	
Pneumocystis jiroveci [number;%]	3; 10%	3; 11%	
Other bacterial pathogen [number;%]	9; 31%	9; 32%	
Fungal Infection^a^ [number;%]	6; 21%	7; 25%	
Viral infection [number;%]	8; 28%	15; 54%	p=0.0526
Influenza [number;%]	3; 10%	10; 36%	p=0.0295
Herpes Virus [number;%]	6; 21%	9; 32%	
Unknown pathogen [number;%]	9; 31%	3; 11%	
Days of antibiotic therapy	12/ 2/ 40	18/ 3/ 65	p=0.0096
Extrapulmonary Cause [number;%]	7; 24%	1; 4%	
ECMO therapy			
Duration [days; median/ min/ max]	14/ 1/ 45	18/ 4/ 78	p= 0.0582
Days until reduction [median, min, max]	10/ 6/ 22	16/ 4/ 42	p= 0.1106
Extrapulmonary Organ Failure			
Days of Vasopressors [median, min, max]	12/ 2/ 27	13,5/ 2/ 24	p=0,1603
Renal Replacement Therapy [number; %]	19; 66%	15; 54%	p=0.36

^a^Fungal infection including all organ systems

Five patients in the control group suffered ARDS of extrapulmonary origin (three patients with pancreatitis and two with polytrauma). The initial PaO_2_/FiO_2_ ratio in the IVIG group was significantly lower than that in the control group (IVIG, median 67 vs control, 93; *t* test, log-transformed data, *p* = 0.009). Seventy-five percent of the IVIG-treated patients had severe ARDS, 25% had moderate ARDS, and no patient showed mild ARDS. In the control group, 55% of patients had severe ARDS, 35% had moderate ARDS, and 10% had mild ARDS. The ECMO duration was shorter in the control group (1-45 days; median 14 days) compared to that in the IVIG group (4–78 days; median 18 days) and could be reduced earlier in the control group (median 10 days) than in the IVIG group (median 16 days). The duration of ventilator support was longer in the IVIG group (91–2005 h; median 571 h) compared to that in the control group (20–1323 h; median 399 h; *t* test, log-transformed data, *p* = 0.055). Five patients in the control group and three patients in the IVIG group were extubated before ECMO was discontinued. Ten patients in the control group and seven patients in the IVIG group suffered anemia due to bleeding complications, including cerebral hemorrhage (control: *n* = 3; IVIG: *n* = 2) (Table 1).

All patients suffered from septic shock requiring vasopressor therapy (control, 1–27 days vs. IVIG, 2-42 days). Renal replacement therapy was more often required in the control group (54% vs. 66%; *p* = 0.36; Pearson’s chi-squared test). Hepatobiliary dysfunction was comparable between both groups (control, 24%; IVIG, 21%).

### Infections

In 66% of the control patients and 82% of the IVIG-treated patients, one or more pathogens could be identified as the cause of pulmonary inflammation. In 11 (38%) patients in the control group, bacterial pathogens such as *Legionella* (*n* = 1; 3%), *Streptococcus pneumoniae* (*n* = 1; 3%), *Pneumocystis jirovecii* (*n* = 3; 10%), and *E. coli* (*n* = 3; 10%) were identified in BAL samples. None of the control patients showed multidrug-resistant bacteria in any samples. In 28% (8 of 29) of the control patients, a viral pathogen was detected in BAL samples: influenza (*n* = 3; 10%) and herpes virus (*n* = 6; 21%). Herpes virus infection included HSV (*n* = 5; 17%) and CMV (*n* = 2; 7%). Herpes viral infection was confirmed by cell-based culture. In five of the control patients, fungal infection was diagnosed via blood culture (*n* = 1), abdominal sample (*n* = 2), and BAL (*Aspergillus*, *n* = 2). In nine control patients, no causal pathogen could be identified (31%).

Two of the 28 IVIG patients were infected with resistant bacteria (3MRGN *Stenotrophomonas maltophilia* and MRSA). In 11 IVIG patients, bacterial pathogens such as *Legionella* (*n* = 3; 11%), *Streptococcus pneumoniae* (*n* = 3; 11%), *Pneumocystis jirovecii* (*n* = 3; 11%), and *Mycoplasma* (*n* = 1) were identified in BAL samples. Viral infections were more frequent in the IVIG group (*p* = 0.046; Pearson’s chi-squared test), especially influenza infection (*p* < 0.05). In more than half of the IVIG patients, viral pneumonia was diagnosed according to the results of BAL samples, radiological findings, and clinical symptoms. One or more of the following viruses could be detected in BAL samples: influenza (*n* = 10; 36%) and herpes virus (*n* = 9; 32%). Herpes virus infection included HSV (*n* = 7; 25%), CMV (*n* = 2; 7%), and HHV6 (in BAL and blood; *n* = 1). In 7 of the IVIG-treated patients, fungal infection was diagnosed in urinary tract (*n* = 4) and abdominal samples (*n* = 2). In addition, one patient suffered aspergillosis pneumonia. In three IVIG-patients, no causal pathogen could be identified (11%).

The duration of anti-infective treatment was significantly longer in the IVIG group than that in the control group (control, median 12 days; 2–40 days; IVIG, median 18 days; 3–65 days; *t* test, log-transformed data, *p* = 0.0096). Six patients in the control group and four patients in the IVIG group underwent anti-infective treatment before admission to the ICU. Excluding these patients, the duration of anti-infective therapy relative to LOS in the ICU was not significantly different between the groups (control median, 71% fraction of LOS ICU; IVIG, 79% of LOS ICU).

### Outcomes

**Fig. 1:**
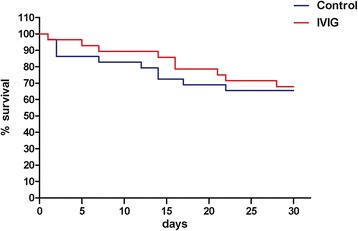
Survival curve of patients who received intravenous IGM therapy and control patients

Patients treated with IVIG stayed for a median of 24.5 days in the ICU (5 to 89 days) and 28.5 days in the hospital (5 to 92 days) (Table 2, Fig. 1). Forty-three percent (12 out of 28) of these patients died; three patients died within the first week due to cerebral hemorrhage (*n* = 2) or multiorgan failure due to septic shock (*n* = 1). The control patients spent a median of 24 days in the ICU (1 to 56 days) and 27 days in the hospital (1 to 88 days); 52% (15 out of 29) of these patients died. Five patients died during within first week due to cerebral hemorrhage, heart failure, subarachnoidal bleeding (polytrauma), and hypoxic brain injury 24 h after admission, and others died of multiorgan failure due to acute pancreatitis.

**Table 2:** Outcome of study patients

Outcome	ARDS patients control	ARDS patients *with* IV IgM	*p* value
Mortality [number; %]	15/52%	12/43%	> 0.5
LOS ICU [median, min, max]	24/1/56	24.5/5/89	0.096
LOS hospital [median, min, max]	27/1/88	28.5/5/92	0.16

### Lymphocyte dynamics in ARDS patients treated with IVIG and in control patients

**Fig. 2:**
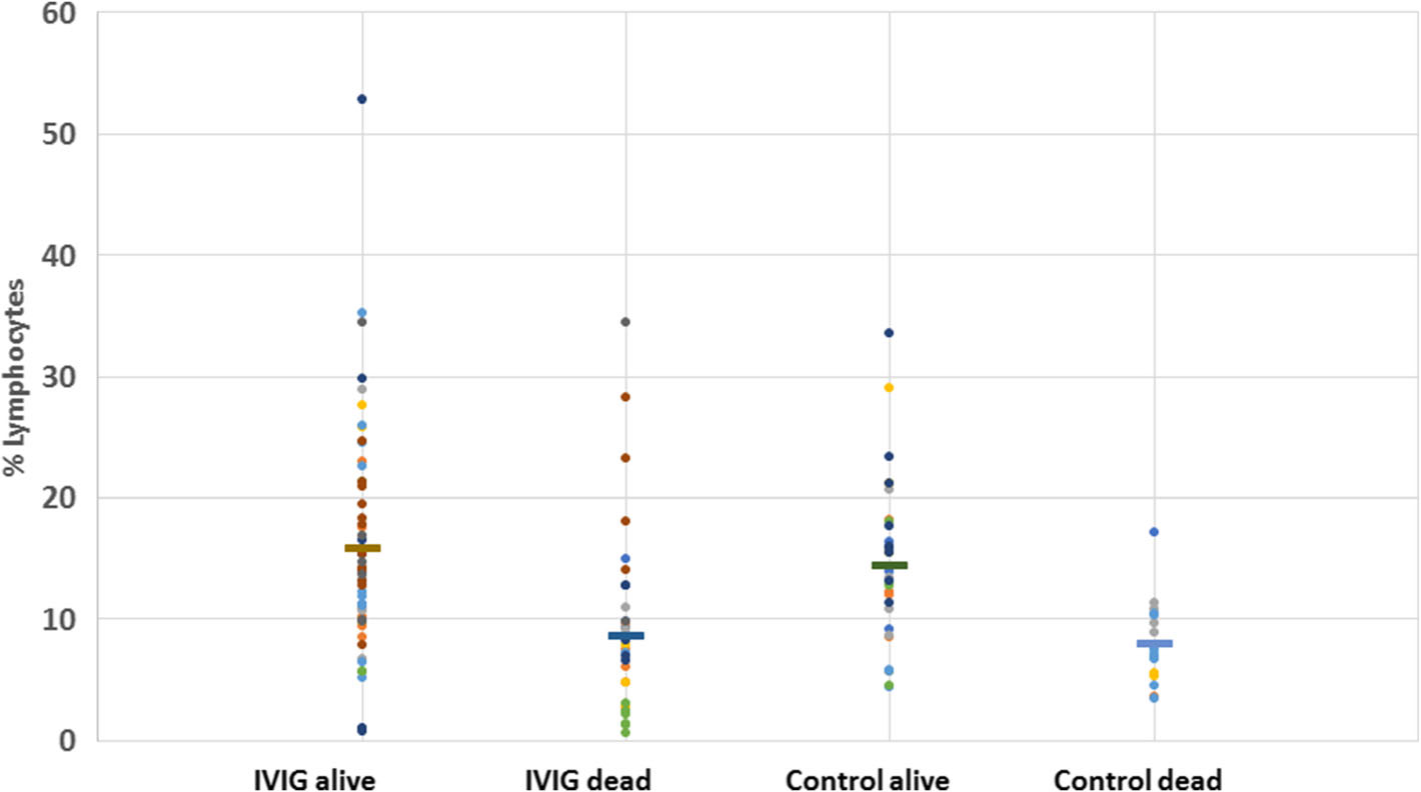
Lymphocyte levels in control patients and patients treated by IVIG during treatment

**Fig. 3:**
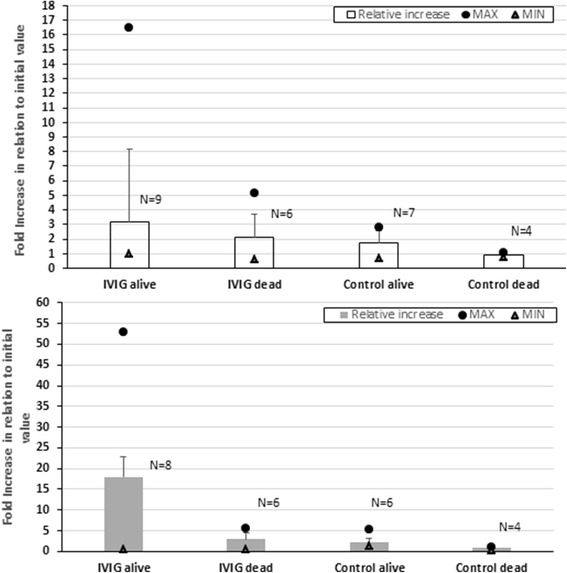
Maximal fold increase in relation to initial value after 10 days (top panel) and 28 days (lower panel). Maximal and minimal values are indicated by dots

Lymphocyte levels were analyzed retrospectively when available. Initially after admission, lymphocyte levels were not different between the groups (control alive 8.3% ± 3.4; control dead 8.9% ± 4.5; IVIG alive 8.9% ± 4.4; IVIG dead 7.3% ± 5.7). However, lymphocyte levels were higher in survivors compared to those in non-survivors (Fig. 2). The increase in lymphocytes was more prominent in survivors compared to that in non-survivors (control alive 1.74 ± 0.85; IVIG alive 3.16 ± 5.03; control dead 0.92 ± 0.17; IVIG dead 2.13 ± 1.55; Fig. 3). In addition, in patients treated with IVIG, this increase during treatment was more prominent than that in the control group in the first 28 days (Fig. 3). Since we did not evaluate differential blood counts routinely, limited data were available for determining significant difference.

## Discussion

The purpose of this analysis was to systematically investigate the potential effect of IgM-enriched immunoglobulins on the outcomes of ARDS patients requiring ECMO therapy. We analyzed 57 patients; 28 patients were treated with IgM-enriched IVIG, and 29 patients did not receive IVIG therapy. Of all patients suffering from septic shock, 75 % of the IVIG-treated patients and 55% of the control patients had severe ARDS of various origins. However, although the IVIG group had a lower PaO_2_/FiO_2_ ratio and required longer ventilation support, longer vasopressor therapy, and longer anti-infective therapy and included MRGN-infected patients, outcome parameters such as LOS in the ICU, LOS in the hospital, and mortality were not significantly different between the groups.

In several studies, hypogammaglobulinemia and over-expression of genes encoding immunoglobulin segments were identified in patients with septic shock [6, 16, 17]. Therefore, IVIG treatment could be a logical requirement in these patients. We included IVIG therapy in our ARDS patients based on these considerations, although we did not measure immunoglobulin levels before treatment. Indeed, in a recent retrospective analysis of 543 patients, high IgG levels were associated with high mortality in sepsis patients [18]. PCR analysis showed an early molecular response to IgM in the blood of patients with sepsis [19]. IgM levels remain low in non-surviving sepsis patients, whereas IgM levels increase transiently in surviving patients [20]. Single studies and meta-analyses have shown beneficial effects of IVIG therapy in patients with sepsis [21–23]. However, IVIG treatment remains controversial. Randomized studies have not shown any beneficial effect of this intervention in patients with sepsis or sepsis-associated conditions [8]. Therefore, IVIG therapy is not currently recommended in the latest sepsis guidelines [24].

Immunoglobulins interact with CD4 T-lymphocytes during bacterial eradication. In severe sepsis, B and T cells are depleted due to apoptosis [25]. Since our analysis is retrospective, we could not measure HLA-DR expression or cytokine profiles in these patients. However, we found a trend of increased lymphocytes in non-survivors compared to survivors, which may reflect why we did not see any beneficial effect of IVIG therapy. Although immunoglobulins may play an important role in the innate immune response during ARDS, non-specific immunoglobulins may not be as effective in a lymphopenia environment. Lymphopenia has been described in ARDS patients and in animal models after viral infection or mycoplasma infection and affects all lymphoid tissue [26–28]. However, in our small group of non-survivors, the percentage of lymphocytes rather than impaired kinetics over time was impressive. Recent studies have shown that patients with ARDS may respond differently to various therapy regimens [29, 30], but whether this variability in response is due to genetic alterations [31], immune evasion by bacteria [32], or different stages of sepsis is unknown.

This study has some limitations. It is a retrospective analysis of a limited number of ARDS patients. Since we changed our ECMO system during the study period, we only included patients treated with the one system to exclude any impact of system differences. Furthermore, since IVIG was ordered by a physician primarily when a viral infection or MRGN infection was suspected, the IVIG group included more patients with these infections. However, this may reflect true clinical circumstances in that the cause of disease is typically not determined at admission.

## Conclusion

ARDS is a multifactorial disease with a heterogeneous pathogenesis and variable timing and clinical presentations. Therefore, one specific therapy is unlikely to improve outcomes. Rather than excluding single therapeutic options, identifying patients’ risk factors and the individual ARDS stage may be more important for successful outcomes. We report here in our retrospective analysis that intravenous IgM administration in the initial stages of severe ARDS did not improve overall outcomes or the severity of disease.

## Abbreviations

APACHE: Acute Physiology and Chronic Health Evaluation
ARDS: Acute respiratory distress syndrome
BAL: Bronchoalveolar lavage
CFU: Colony-forming unit
CMV: Cytomegalovirus
ECMO: Extracorporeal membrane oxygenation
ELSO: Extracorporeal Life Support Organization
FiO_2_: Inspiratory oxygen concentration
HLA-DR: Human leukocyte antigen–antigen D-related
HSV: Herpes simplex virus
ICU: Intensive care unit
Ig: Immunoglobulin
IVIG: Intravenous immunoglobulin
LOS: Length of stay
MRGN: Multi-resistant gram-negative bacteria
MRSA: Methicillin-resistant *Staphylococcus aureus*
PaO_2_: Arterial oxygen partial pressure
PCR: Polymerase chain reaction
SAPS: Simplified Acute Physiology Score
